# Extracellular field signatures of CA1 spiking cell assemblies during sharp wave-ripple complexes

**DOI:** 10.1186/1471-2202-14-S1-O14

**Published:** 2013-07-08

**Authors:** Jiannis Taxidis, Kamran Diba, Costas A Anastassiou, György Buzsáki, Christof Koch

**Affiliations:** 1 Computation and Neural Systems, California Institute of Technology, Pasadena, CA 91125, USA; 2 Department of Psychology, University of Wisconsin at Milwaukee, Milwaukee, WI 53201, USA; 3 NYU Neuroscience Institute, New York University, New York, NY 10016, USA

## 

Although postsynaptic and transmembrane currents over local neuronal populations are considered the main factors for shaping local field potential (LFP) and current source density (CSD) fluctuations [1], high-frequency oscillatory LFPs can also be shaped by extracellular action potentials of pyramidal cell populations [2]. Sharp wave-ripple complexes (SWRs) are typical examples of such high-frequency oscillatory events, observed in hippocampal LFPs during deep sleep and awake immobility. They consist of an extensive depolarization in the CA1 dendritic layer (sharp wave) arising from population bursts in CA3, accompanied by a ~150-200 Hz LFP oscillation in the CA1 pyramidal layer (ripple). During SWRs, temporal firing patterns of correlated place cells, acquired during wakeful exploration, are replayed in fast-scale, providing a strong indication for the participation of SWRs in memory consolidation. Yet the particular effects of these pattern replays on the hippocampal extracellular field are largely unknown. How are the different ensembles of spiking cells encoded in the emerging ripple-LFPs? Here, we study this association through both a modeling and an experimental approach.

Firstly, we employ a spiking network model of the CA3 and CA1 hippocampal areas that reproduces key features of SWRs based on synchronous CA3 population bursts and strong, fast-decaying CA1 recurrent inhibition [3]. The synaptic input on CA1 pyramidal cells is implemented in a population of morphologically realistic, multi-compartmental models of CA1 pyramidal neurons, based on reconstructed cells [4]. The emerging extracellular fields accurately reproduce properties of SWR LFPs. By developing different spatial distributions of sets of CA1 cells that fire during ripples and others that remain silent, we explore in a systematic fashion the influence of spiking cell assemblies on the spatiotemporal characteristics of emerging extracellular fields during SWRs. In particular, we show how the different spatial arrangements of spiking cells give rise to differences in the depth-profile and CSD characteristics of raw and filtered LFPs.

Next, we apply our analysis to a set of LFPs and unit activity, recorded *in vivo*, from multiple locations in areas CA3 and CA1 of the rat hippocampus while animals run on a linear track with resting areas at both ends. The spiking activity of detected place cells, firing in sequence during the running sessions, is replayed in either forward or reverse order during SWRs occurring at the resting areas [5]. We trace the differences in the depth profile and CSDs of SWR LFPs between such forward and reverse replays. Based on our modeling results, we provide a link between systematic changes in the spatiotemporal features of the LFPs, with the corresponding ensembles that gave rise to each ripple episode.

This work provides a deeper understanding of the nature of extracellular fields and offers a new approach to the decoding of ongoing cell assemblies based on extracellular current flows. Differences in the emerging SWR field activity may play an important role in information processing during memory consolidation.
